# The SALINE Technique for the Treatment of the No-Reflow Phenomenon during Percutaneous Coronary Intervention in STEMI

**DOI:** 10.3390/jcm12062405

**Published:** 2023-03-21

**Authors:** Luca Grancini, Davide Diana, Alice Centola, Giovanni Monizzi, Angelo Mastrangelo, Paolo Olivares, Piero Montorsi, Brunilda Alushi, Antonio L. Bartorelli, Alfredo R. Galassi

**Affiliations:** 1 Ospedale Galeazzi Sant’Ambrogio, IRCCS, 20157 Milan, Italy; 2 Department of Promise, University of Palermo, 90133 Palermo, Italy; 3 Centro Cardiologico Monzino, IRCCS, 20138 Milan, Italy; 4 Department of Clinical Sciences and Community Health, University of Milan, 20122 Milan, Italy; 5 Department of Cardiology, Campus Benjamin Franklin, Charite’ Medical University Berlin, 12203 Berlin, Germany; 6 Department of Internal Medicine, Cardiology and Angiology, Zollernalb Klinik Balingen, 72336 Balingen, Germany; 7 Department of Biomedical and Clinical Sciences “Luigi Sacco”, University of Milan, 20122 Milan, Italy

**Keywords:** STEMI, no-reflow, SALINE technique

## Abstract

Background: Primary percutaneous coronary intervention (pPCI) performed for STEMI may be complicated by the “no-reflow” phenomenon. Aims: A super-selective intracoronary injection of saline solution through a thrombus aspiration catheter (SALINE technique), was investigated for the treatment of no-reflow as compared with the standard care of therapy (SCT). Methods: Among the 1471 patients with STEMI undergoing pPCI between May 2015 and June 2020, 168 patients developed no-reflow. Primary endpoints were the incidence of ST-segment resolution (STR) ≥ 70% at 90 min after PCI and the rate of flow restoration (TIMI flow grade 3 with an MBG > 1). The secondary endpoint was the incidence of major adverse cardiac and cerebrovascular events at 3 years follow-up. Results: After propensity score matching analysis, patients treated with SALINE showed STR ≥ 70% in twelve out of the sixteen patients (75.0%), compared to only three patients out of the sixteen in the SCT control group (19.0%), (*p* < 0.004). SALINE was associated with a higher probability of final TIMI flow grade 3 with an MBG > 1, as shown in fourteen out of sixteen patients (87.5%), as compared to only seven out of sixteen patients in the SCT group (43.8%), (*p* < 0.03). MACCE at 3 years follow-up occurred in only one patient (6.3%) in the SALINE group, as compared to eight patients (50%) in the SCT group (*p* = 0.047). Conclusions: The SALINE technique showed to be a safe and effective strategy to reduce “no-reflow” in STEMI patients as assessed by significant STR, improvement of TIMI flow grade, and better 3-year outcomes.

## 1. Introduction

The prompt reopening of the infarct-related artery by primary percutaneous coronary intervention (pPCI) is the main therapeutic goal in patients with ST-segment elevation myocardial infarction (STEMI) [1]. However, in 30–50% of STEMI, pPCI may be complicated by microvascular obstruction (MVO), defined as inadequate myocardial perfusion despite successful recanalization of the infarcted related artery [2,3]. After pPCI, MVO may be postulated if, in the absence of any PCI-related complications of epicardial vessels, ST resolution (STR) within 60–90 min is <70%, and/or thrombolysis in myocardial infarction (TIMI) flow is <3, and/or in the case of a TIMI flow 3 myocardial blush grade (MBG) of 0–1 [1].

MVO represents a major predictor of poor clinical outcomes [4] and, over the years, several studies investigated how to prevent and treat this complication [5,6,7,8,9,10,11,12,13]. Several strategies targeting ischemia-reperfusion injury have been tested by clinical and randomized studies and, despite some of them having shown a certain benefit, especially when more than one strategy was adopted at the same time, none of these studies showed significantly better outcomes than the others.

Considering these contradictory results on the resolution of MVO [13], ESC guidelines do not recommend a specific treatment strategy for treating this complication after STEMI and pPCI, leaving a major gap in decision-making when no-reflow occurs [1,14].

The aim of the present study was to prove the safety, feasibility, and effectiveness of the SALINE technique to restore myocardial perfusion compared with the standard care of therapy (SCT) in case of no-reflow after pPCI. The SALINE technique consists of a super-selective forceful manual injection of a 10 mL bolus of saline through a thrombus aspiration catheter in a coronary artery with no-reflow right beyond the site of the pressure drop.

## 2. Materials and Methods

### 2.1. Study Design and Definitions

This is a “pilot” observational study to assess the safety, feasibility, and effectiveness of the SALINE technique. It was led prospectively at a single centre based on a real cohort of patients with STEMI who underwent revascularization by pPCI between May 2015 and June 2020. Informed consent was obtained on admission for each patient enrolled. Inclusion criteria were the onset of symptoms (chest pain or equivalent) ˂12 h before the first medical contact, ST-segment elevation of at least 2 mm in 2 or more contiguous leads, and TIMI flow 0–2 at baseline angiography. Patients were pretreated with dual antiplatelet therapy with aspirin (300 mg loading dose and 100 mg maintenance dose) and either prasugrel (60 mg loading dose and 10 mg maintenance dose), ticagrelor (180 mg loading dose and 90 mg twice daily maintenance dose), or clopidogrel (600 mg loading dose and 75 mg maintenance dose), followed by unfractionated heparin 5000 IU, according to the latest European guidelines [1,15]. Echocardiogram was performed at admission to assess the left ventricle ejection fraction. PPCI was performed through radial or femoral access using 6 or 7 Fr sheaths. A twelve-lead electrocardiogram (ECG) was recorded at arrival and at each angiography time point to evaluate the degree of STR. Angiographic images were acquired at 30 frames per second with long acquisitions (to better visualize the venous phase in contrast passage) in orthogonal views before intervention and after stenting (at the time of the final/optimal angiographic result) to enable the determination of angiographic markers of MVO offline at our core laboratory. TIMI flow grade assessment and digital quantification of MBG were performed as previously described [16,17].

After manual thrombectomy, when applied, a bolus of nitroglycerin 200 μg was injected as distally as possible via the thrombus aspiration catheter.

Following stent deployment, MVO was diagnosed if, in the absence of PCI-related complications of epicardial vessels (spasm, flow-limiting dissection, incomplete lesion dilation, stent under-expansion, stent fracture, stent thrombosis, or distal embolization of plaque/thrombus debris), ST resolution (STR) within 60–90 min was <70% and/either TIMI flow was <3, or in the case of TIMI flow 3, MBG was 0–1 [1].

In the case of MVO, intracoronary repeated boluses of adenosine were administered through the guiding catheter. Thereafter, according to the operator’s preferences, either the SALINE technique or SCT was chosen as the treatment strategy. The SALINE technique is described in detail in the next paragraph. SCT was performed by administering an intracoronary bolus of 200 μg of nitrates and, according to thrombotic burden and operator preferences, with an intracoronary slow bolus injection (>1 min) of Abciximab 0.25 mg/kg.

An intra-aortic balloon pump (IABP) according to clinical and haemodynamic conditions was used if indicated in both groups. Cardiac enzymes were checked at 4, 12, and 24 h after the pPCI and a 12-lead ECG was recorded 90 min and 24 h after the procedure and before discharging the patient. Naïve patients were commenced on beta-blockers, angiotensin-converting enzyme (ACE) inhibitors, high-dose statins, and dual antiplatelet therapy, unless contraindicated, according to international guidelines [1].

The institutional Ethics Committee approved the data collection and analysis of the study. All patients were followed-up for at least 3 years following the pPCI.

### 2.2. The SALINE Technique

**Figure 1:**
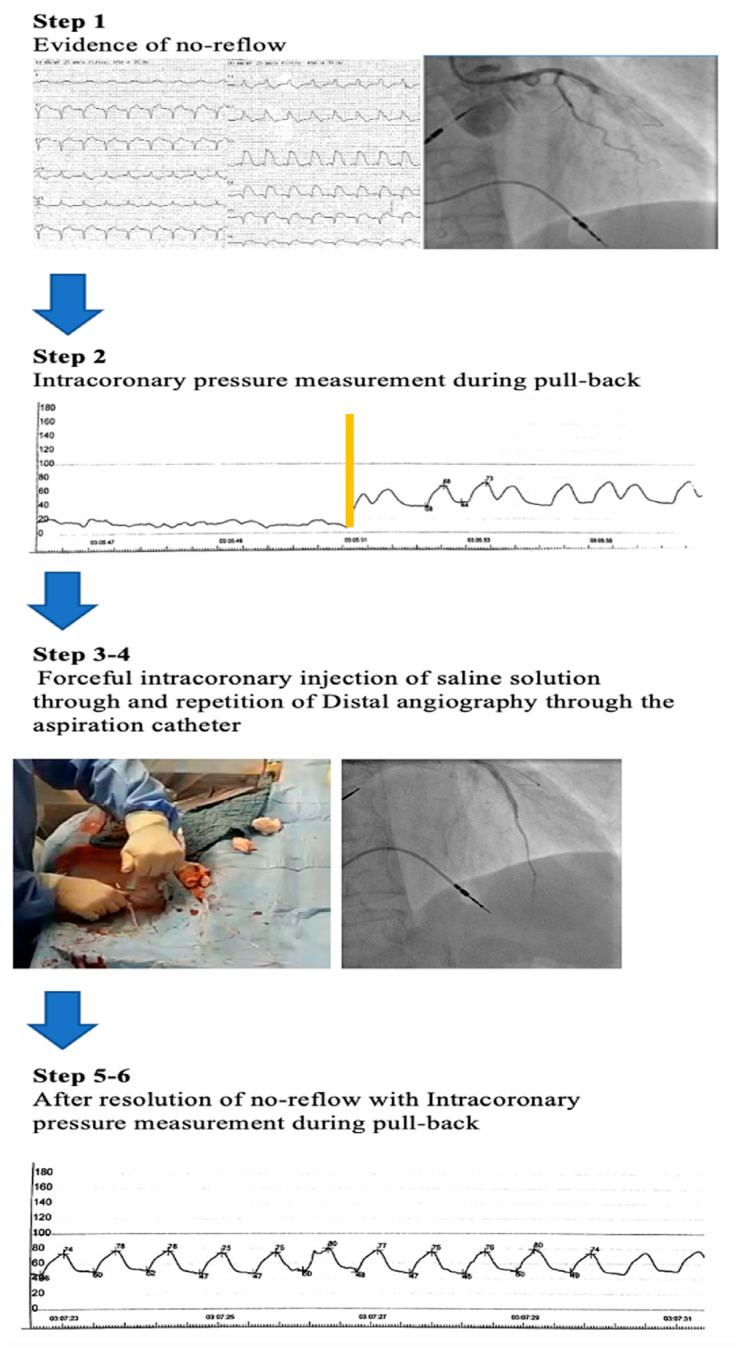
Stepwise flowchart of a successful patient treated using SALINE. The figure describes a case of an anterior STEMI secondary to left anterior descending artery (LAD) thrombotic occlusion after evidence of no-reflow (see text for an explanation of all different steps). Step 2. The yellow line represents the site of intracoronary pressure drop measurement, recorded from the distal to the proximal coronary vessel. Step 3. A forceful intracoronary injection of a 10 mL bolus of saline solution over 3 to 4 *s* through the thrombus aspiration catheter was performed. Step 4. Distal angiography through the aspiration catheter was repeated to assess TIMI flow; in case of persistence of no-reflow, the last step was repeated sequentially 2–3 times Step 5. On the left panel is the ECG recording and on the right panel is the conventional coronary angiography after the resolution of the no-reflow phenomenon. Step 6. Intracoronary pressure measurement recorded after the resolution of no-reflow from the distal to the proximal coronary vessel. No evidence of pressure drop was registered.

The SALINE technique consisted of a super selective and forceful manual injection of saline through the thrombus aspiration catheter in the vessel affected by no-reflow. The stepwise approach of the SALINE protocol is summarized in Figure 1. After evidence of no-reflow at conventional angiography, the SALINE technique was performed as follows. (1) Distal angiography, as defined by a super selective injection of contrast media through a thrombus aspiration catheter, was performed beyond the point of interrupted flow in case of TIMI 0 flow, or at mid coronary artery segment in case of TIMI 1–2 flow or normal TIMI 3 but MBG 0–1 (Step 1). (2) The thrombus aspiration catheter was then connected to the external transducer to measure the pressure at its tip during a pullback maneuver. (3) An intracoronary pressure measurement was recorded from the distal to the proximal coronary vessel to properly identify the best site of angiographic pressure drop (Step 2). (4) A forceful manual intracoronary injection of a 10 mL bolus of saline solution over 3 to 4 s through the thrombus aspiration catheter was performed (Step 3). (5) Distal angiography was repeated to assess TIMI flow; in case of persistence of no-reflow, the last step was repeated sequentially 2–3 times (Step 4). Finally, conventional coronary angiography was repeated (Step 5) and intracoronary pressure measurement was recorded from the distal to the proximal coronary vessel by performing another pullback maneuver to assess the efficacy of the treatment (Step 6).

### 2.3. Study Endpoints

The primary endpoint was the rate of ST-segment resolution (STR) ≥ 70% at 90 min after PCI [18] and the rate of coronary restoration flow, defined as the percent of patients with a TIMI 3 flow and with an MBG > 1.

The secondary endpoint was the rate of major adverse cardiac and cerebrovascular events (MACCE), a composite of cardiovascular death, myocardial infarction, target-lesion revascularization (TLR), and heart failure requiring hospitalization at 3 years follow-up. Myocardial infarction was defined according to the “Fourth definition of myocardial infarction” [19] and TLR was defined as repeated revascularization of a lesion through a redo-PCI or CABG surgery. Furthermore, the rate of combined MVO (concordance or discordance between STR and angiographic MVO) was compared between groups.

Additional outcomes were considered, such as (1) the combined STR and angiographic MVO resolution; (2) left ventricle ejection fraction at discharge; (3) in-hospital peak value of Hs-TnI; (4) bleeding; and (5) the left ventricle ejection fraction delta variant at 1-year follow-up. Hs-TnI was the marker of choice to assess myocardial injury [1]. Procedural safety was assessed based on the occurrence of an atrio-ventricular block (transient or requiring pacing), hypotension requiring intra-aortic-balloon-pump, ventricular tachycardia or fibrillation, and CV death.

### 2.4. Statistical Analysis

To minimize potential selection bias in the SALINE group, a propensity score matching with a 1 to1 ratio was performed with the nearest neighbor method for the following variables: age, gender, diabetes, onset symptoms to wire delay, ST-segment elevation, cardiogenic shock, culprit vessel, number of diseased vessels, TIMI flow grade, and treatment with diuretics.

Categorical variables are presented as counts and percentages and compared by Fisher’s exact test. Continuous variables are presented as the median and inter-quartile range (IQR) or mean and standard deviation and compared by the Mann–Whitney U test or the Unpaired *t*-test. The effectiveness endpoints (coronary flow restoration rate and complete STR) were compared between groups with Fisher’s exact test. Concerning the combination of STR and angiographic MVO resolution, a 3-point score was adopted, ranging from 0 to 2 based on whether none, one, or both endpoints were met, and was compared between groups with the Mann–Whitney U test.

For such a pilot study, we considered a minimum sample size of 12 patients per group [20]. All tests were 2-tailed, and a *p*-value of <0.05 was considered statistically significant. All analyses were performed using SAS version 9.4 (SAS Institute, Cary, NC, USA).

## 3. Results

### 3.1. Study Population

**Figure 2:**
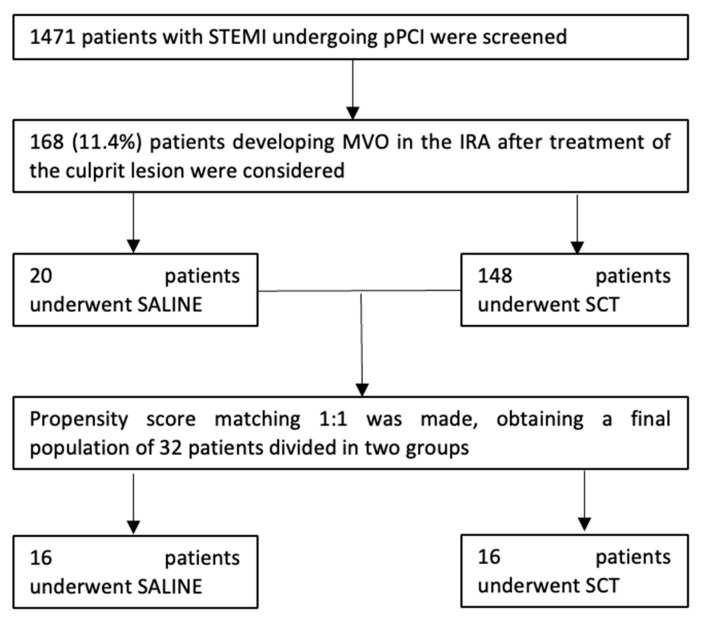
Population selection flowchart. IRA = infarct related artery; MVO = microvascular obstruction; pPCI = percutaneous primary coronary intervention; SALINE = saline-induced coronary hyperemia technique; SCT = standard care of therapy; STEMI = ST-elevation miocardial infarction.

Out of 1471 patients with STEMI undergoing pPCI, 168 patients (11.4%) had MVO in the infarcted-related artery after treatment of the culprit lesion and were included in the study. Among these patients, 16 were treated with the SALINE technique and were matched with 16 patients who were treated with the SCT. The final population consisted of 32 patients: 16 patients underwent the SALINE and 16 patients underwent the SCT strategy (Figure 2).

The baseline characteristics of patients in both groups did not show any statistical difference (Table 1). Angiographic and procedural data are summarized in Table 1 and Table 2. Most of the patients in both groups showed TIMI flow 0, while the minority of them showed TIMI flow 1–2 and no one showed TIMI flow 3. A total of 11 patients (68.8%) in the SALINE group and 12 patients (75.0%) in the SCT group received adenosine (*p* = NS). Nitrates were administered in all patients of the SCT group and abciximab was administered in only two patients (13.3%) in SCT group (*p* = 0.913). A total of four patients, two in SALINE two in SCT, developed hypotension not requiring the use of a mechanical assistance device, while IABP was deemed necessary in three patients in SALINE and two patients in SCT (18.8% vs. 12.5%, respectively, *p* = NS), New onset AV block, not any requiring pacing, occurred in four (25%) patients in the SALINE group and two patients (12.5%) in the SCT group (*p* = 0.654). Ventricular arrythmias, such as ventricular tachycardia, occurred only in one patient in SCT (Table 3).

### 3.2. Primary Endpoint and In-Hospital Outcomes

**Figure 3:**
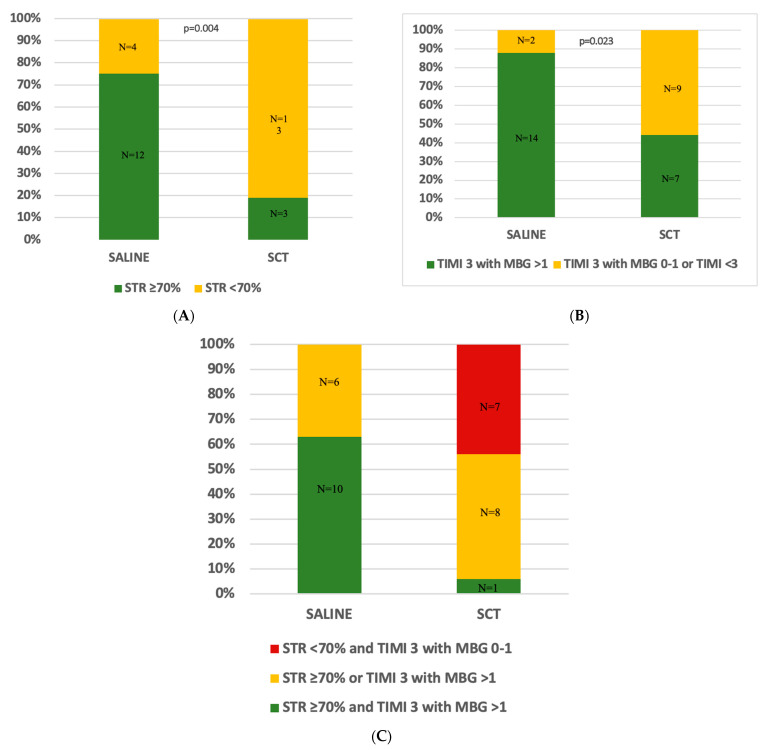
Myocardial reperfusion data were assessed by TIMI flow and STR according to the treatment group. Panel (**A**). The percentages of patients are shown according to the degree of ST-segment resolution (STR): STR < 70% or STR ≥ 70%. Panel (**B**). The percentages of patients are shown according to the final TIMI flow grade 3 with myocardial blush grade (MBG) > 1 vs. TIMI 3 with MBG 0–1 or TIMI < 3. Panel (**C**). The percentages of patients are shown according to the positive concordance of STR > 70% and TIMI 3 with MBG > 1, discordance between STR ≥ 70% and TIMI 3 with MBG > 1, and negative concordance between STR < 70% and TIMI 3 with MBG 0–1 or TIMI < 3. Abbreviations: MBG = myocardial blush grade; SALINE = saline-induced coronary hyperemia technique; SCT = standard care of therapy; STR = ST segment resolution; TIMI = thrombolysis in myocardial infarction.

Data regarding myocardial reperfusion and in-hospital clinical outcomes are shown in Table 4. STR ≥ 70% was observed in twekve patients (75%) in the SALINE group, but only in three patients (18.8%) in the SCT group (*p* = 0.004) (Figure 3A). Moreover, final TIMI flow 3 and MBG > 1 were achieved in fourteen (87.5%) patients in the SALINE group and only in seven (43.8%) in the SCT group (43.8%) (*p* = 0.023) (Figure 3B). Combined STR and angiographic MVO resolution was observed in ten patients (62.5%) in the SALINE group and one patient (6.3%) in the SCT group (*p* < 0.001) (Figure 3C). Concerning the combination of resolution of STR and angiographic MVO, an increasing trend was shown in the SALINE group compared with the SCT group (*p* = 0.0002 MH chi-square). A total of ten patients in the SALINE group (63.5%) and one patient in the SCT group (6.3%) reached both endpoints; six (37.5%) and eight (50%) in the SALINE and the SCT group, respectively, which resolved one of the two, while seven patients did not meet either outcome (n = 7; 43.8%), and all were from the SCT group. The rate of concordance or discordance between STR and angiographic MVO is shown in Figure 3C.

Left ventricle ejection fraction at discharge and peak of Hs-TnI did not show statistically significant differences between the SALINE and SCT groups (52.9% ± 8.8% vs. 55.6% ± 11.4%, *p* = 0.478, 76.821 ± 69.402 ng/dL vs. 79.989 ± 78.261 ng/dL, respectively, *p* = 0.720).

### 3.3. Secondary Endpoints

MACCE occurred in one patient (6.3%) in the SALINE group and eight patients (50.5%) in the SCT group (*p* = 0.047). CVE occurred in none of the patients (6.3%) in the SALINE group and two patients (12.5%) in the SCT group. In only one patient (6.3%) in the SCT group was there minor and self-limiting bleeding event that occurred. No cardiovascular death events were observed in both groups, and only one patient in the SCT group had a myocardial infarction. No TLR was observed in the SALINE group, while two patients (12.5%) underwent TLR in the SCT group. HF hospitalizations occurred in one patient (6.3%) in the SALINE group and three patients (18.8%) in the SCT group.

## 4. Discussion

The present study shows that a super-selective manual forceful injection of saline solution through a thrombus aspiration catheter (SALINE technique) is safe and effective in restoring myocardial perfusion in case of no-reflow after pPCI. The effectiveness of this technique is reflected by either a rapid ST-segment resolution, or normalization of final TIMI flow in the majority of patients.

The ischemia/reperfusion injury, MVO, and final infarct size are major independent predictors of long-term mortality and heart failure after STEMI [2,21]. MVO is diagnosed immediately after PCI when postprocedural angiographic TIMI flow is <3 or TIMI flow 3 when MBG is ≤1 (angiographic MVO), or when ST resolution within 60–90 min of the procedure is <70%. Although cardiac magnetic resonance (CMR) still remains the gold standard for assessing MVO and infarct size, invasive and flow-related measurements performed immediately after pPCI, such as IMR [22,23,24] and TIMI flow grade [25], showed a good correlation with MVO, as detected by CMR.

The treatment of no-reflow post-pPCI is still challenging and the optimal treatment strategy is controversial, as several clinical and randomized trials comparing different strategies were non-conclusive [3,7,8,9,10,11,12].

A meta-analysis of previous randomized trials demonstrated that the GpIIb/IIIa inhibitor Abciximab as adjunctive therapy could reduce 30-day re-infarctions and mortality in STEMI patients undergoing pPCI, lowering the incidence of MVO after pPCI [7]. Furthermore, as emerged from the randomized TAPAS (thrombus aspiration during primary percutaneous coronary intervention) trial, thrombus aspiration before stenting improved the resolution of ST-segment elevation when compared to standard pPCI [26]. However, in this study the majority of patients (90%) were also treated with Abciximab, suggesting that MVO could be lowered by a combination of thrombus aspiration and GpIIb/IIIa inhibitors. Nevertheless, significant STR was demonstrated only in 50% of these patients and was not associated with a significant reduction in infarct size, suggesting that TA, alone or in combination with Abciximab, was not sufficient to reduce MVO [8]. In the REOPEN-AMI (intracoronary nitroprusside versus adenosine in acute myocardial infarction) trial, Niccoli et al. compared high-dose intracoronary administration of adenosine to nitroprussiate to assess prevention of MVO in STEMI patients treated with pPCI, thrombus aspiration, and Abciximab within 12 h from the onset of symptoms. As a control group, the authors considered patients receiving only heparinized saline bolus after thrombus aspiration and Abciximab. They demonstrated that intracoronary administration of adenosine, but not nitroprussiate, safely improved microcirculatory perfusion, as assessed by the resolution of ST-segment elevation, which showed to be greater in patients with early onset of symptoms [11]. Conversely, the randomized REFLO-STEMI trial based on the use of intracoronary adenosine or nitroprussiate has suggested not only a lack of beneficial effects of both drugs alone but rather the potentially detrimental effects of intracoronary adenosine infusion. Indeed, a significant increase in infarct size and myocardial volumes as assessed by CMR was associated with a trend of reduced left ventricle ejection fraction, leading to an adverse mid-term clinical outcome [12]. Based on these results, the authors strongly discouraged clinicians from using a high dose of intracoronary adenosine during pPCI to prevent reperfusion injury. Another recent multicenter observational study, the RESTORE (efficacy and safety of intracoronary epinephrine versus conventional treatments alone in STEMI patients with refractory coronary no-reflow during primary PCI) study compared the effect of intracoronary epinephrine on top of SCT to overcome a refractory no-reflow after an extensive STEMI. The authors observed that epinephrine yielded significantly better coronary flow patterns and a higher prevalence of STR ≥ 70% than SC alone (75.0% vs. 28.5%), with a significant 30-day reduction in the composite of death and heart failure and improvement of left ventricle ejection fraction early after pPCI [27]. In some cases, Verapamil administration has been described as a potential option for no-reflow but its effects remain controversial (3–5). It was not administered to any patients in this study, as specified in the methods section.

In our pilot study, the SALINE technique as compared to SCT is able to significantly resolve either STR and angiographic MVO, reducing STR ≥ 70% in 75% and restoring TIMI flow 3 with MBG > 1 in 87.5% of cases and causing a combined MVO resolution in 62.5% of patients. The pathophysiological mechanism of action of this technique is not clear yet but several hypotheses are likely involved in the final effect. The efficacy of SALINE might be based on the clearance effect on interstitial edema, greatly enhanced by forceful, super-selective, and repetitive saline administration that may ultimately lead to a rapid wash-out of osmotically active molecules from the interstitial space [3]. Forceful perfusion might also provoke a myogenic reaction to fluctuations in intraluminal pressure, leading to vasomotor adaptations in coronary tone [28]. Nevertheless, the mechanisms of intracoronary saline-induced hyperemia have never been clarified [29,30]. Recent evidence showed that saline-induced hyperemia through a dedicated intracoronary infusion catheter (RayFlow ^®^) is associated with local intravascular hemolysis; in addition, vasodilatory compounds released locally, such as ATP or NO, are likely ultimately responsible for localized microvascular dilatation [31]. However, the same author demonstrated that this hyperemic effect was not obtained when saline was infused at lower infusion rates or through a single distal hole from the same microcatheter [28,32]; nevertheless, we cannot exclude that the higher infusion pressure obtained by manual injection. the larger size of the thrombus aspiration catheter, as well as the distal exits points of the microcatheter, may lead to a hyperemic response despite the existing differences between the two tools. Intracoronary adenosine administration before SALINE administration might have contributed significantly to these distal small arterioles (˂120 μm) dilatation increasing final myocardial flow. Major tissue pressure and myogenic small vessel control might have also contributed significantly to restoring myocardial blood flow as compared to the metabolic factors of the GpIIb/IIIa inhibitors, which conversely might not influence coronary blood flow restoration after no-reflow. Finally endothelial and neurohumoral factors almost certainly play facilitating or modulating roles in restoring myocardial flow but this is difficult to ascertain in our study after the no-reflow phenomenon.

Clinical outcomes up to 3 years follow-up were included in the study as an important safety consideration but the trial was not powered to detect clinically meaningful differences in MACEs and MACCEs between groups, although a significance was detected for MACCE.

No difference was observed in terms of LVEF at discharge and Tn peak (*p* = NS); this might be related to the low sample size of our study. The effect of this technique on infarct size needs to be evaluated in larger studies.

The advantages in using a technique based on the administration of saline are safety, low expensiveness, and facility application. SALINE use overcomes the side effects of many intracoronary medications, such as epinephrine or other medications, that do not seem properly tolerated, as partially above mentioned [12].

### Limitations of the Study

This study presents some limitations. Firstly, although an appropriate propensity score-matched analysis was performed, the non-randomized nature of the study cannot completely exclude selection bias. Secondly, this is a single-center study with a rather low sample size and, therefore, should be considered “hypothesis-generating”. Third, the study was not powered to detect any difference in terms of clinical events between the two groups. Fourth, as the majority of patients in the SALINE group were pre-treated by intracoronary adenosine, we cannot make a distinction of the effect that this drug had before the SALINE technique application. Fifth, the suggested use of GpIIb/IIIa inhibitors by the new guidelines [1] was not implemented systematically in our study; indeed, our study period interval was before the publications of these new guidelines that highlighted a certain benefit for such pharmacological drugs in resolving the no-reflow phenomenon.

## 5. Conclusions

In summary, the SALINE technique is a promising, simple, safe, and inexpensive strategy to implement in every catheterization laboratory. In STEMI patients treated with pPCI complicated by the “no-reflow” phenomenon, SALINE showed to be a safe and effective strategy to resolve “no-reflow”, as shown by significant STR and the improvement of TIMI flow grade. Randomized control trials are needed to investigate the efficacy and reproducibility of the technique and to assess the potential benefit in terms of better prognostic outcomes.

### Perspectives

Almost half of the patients with STEMI treated with pPCI may be complicated by the no-reflow phenomenon, defined as inadequate myocardial perfusion despite successful revascularization, and associated with adverse clinical outcomes. Several treatment strategies have been studied showing contradictory results leaving a major gap in international guidelines. The SALINE (saline-induced coronary hyperemia), consisting of a super-selective intracoronary injection of saline solution through a thrombus aspiration catheter, is effective in restoring myocardial perfusion, assessed by significant ST-segment resolution, the improvement of TIMI flow grade, and better mid-term prognostic outcomes, compared with the standard care of therapy. Randomized controlled trials are needed to investigate the efficacy and reproducibility of the technique and to assess the potential benefit in terms of better prognostic outcomes.

## Figures and Tables

**Table 1:** Baseline characteristics and angiographic data.

	SALINE (n = 16)	SCT (n = 16)	*p*-Value
Age, years	71.4 ± 15.8	69.8 ± 12.3	0.758
Male gender	11 (68.8%)	11 (68.8%)	1.000
BMI, kg/m^2^	25.8 ± 5.0	25.9 ± 2.9	0.903
Hypertension	12 (75.0%)	10 (62.5%)	0.704
Hyperlipidemia	7 (43.8%)	8 (50.0%)	1.000
Diabetes	3 (18.8%)	2 (12.5%)	1.000
COPD	1 (6.3%)	1 (6.3%)	1.000
CAD	3 (18.8%)	3 (18.8 %)	1.000
Current smoking	4 (25.0%)	1 (6.3%)	0.333
eGFR < 60 mL/min	3 (18.8%)	3 (18.8%)	1.000
Killip class > I	3 (18.8%)	2 (12.5%)	1.000
Time from symptoms onset to balloon, minutes	155 [IQR 96, 344]	228 [IQR 130, 405]	0.597
Cardiogenic shock	2 (12.5%)	1 (6.3%)	1.000
Baseline maximal sum of ST-segment elevation, mm	10 [IQR 3-19]	11 [IQR 3-20]	0.934
LVEF at admission, %	46.0 ± 9.3	49.5 ± 12.2	0.380
Therapy at admission			
Aspirin	5 (31.3%)	3 (18.8%)	0.683
Beta-blockers	5 (31.3%)	7 (43.8%)	0.715
ACE inhibitors	4 (25.0%)	1 (6.3%)	0.330
ARBs	4 (25.0%)	6 (37.5%)	0.703
Statins	5 (31.3%)	4 (25%)	1.000
Culprit lesion			
LAD	8 (50.0%)	8 (50.0%)	1.000
LCX	3 (18.8%)	2 (12.5%)	1.000
RCA	4 (25.0%)	5 (31.3%)	1.000
SVG	1 (6.3%)	1 (6.3%)	1.000
Location of the lesion in the culprit vessel			
Proximal	7 (43.8%)	4 (25.0%)	0.458
Medial/Distal	9 (56.3%)	12 (75.0%)	0.458
Number of diseased vessels			
1-vessel CAD	3 (18.8%)	2 (12.5%)	1.000
2-vessel CAD	6 (37.5%)	8 (50.0%)	0.722
3-vessel CAD	6 (37.5%)	5 (31.3%)	1.000
SVG	1 (6.3%)	1 (6.3%)	1.000
TIMI flow grade at admission			
0	13 (81.3%)	13 (81.3%)	1.000
1	2 (12.5%)	2 (12.5%)	1.000
2	1 (6.3%)	1 (6.3%)	1.000
3	0	0	-
Presence of intracoronary thrombus	15 (93.8%)	11 (68.8%)	0.172

Abbreviations: ACE = angiotensin-converting enzyme, ARBs = angiotensin receptor blockers, BMI = body mass index, CAD = coronary artery disease, COPD = chronic obstructive pulmonary disease, IRA = infarct related artery; LAD = left anterior descendent; LCX = left circumflex artery; LVEF = left ventricular ejection fraction, RCA = right coronary artery; SCT = standard care of therapy; SVG = saphenous venous graft; TIMI = thrombolysis in myocardial infarction.

**Table 2:** Procedural data.

	SALINE (n = 16)	SCT (n = 16)	*p*-Value
Oral drugs administration			
Aspirin	16 (100%)	15 (93.8%)	1.000
Clopidrogel	5 (31.3%)	4 (25.0%)	1.000
Ticagrelor	2 (12.5%)	7 (43.8%)	0.112
Prasugrel	9 (56.3%)	5 (31.3%)	0.285
IV bolus administration			
Cangrelor	0	0	-
Unfractionated heparin (5000 IU)	16 (100%)	16 (100.0%)	-
Abciximab (0.25 mg/kg)	0	0	-
Radial access	12 (75%)	13 (81.3)	1.000
Femoral access	4 (25%)	3 (18.8%)	1.000
Thrombus aspiration	15 (93.8%)	10 (62.5%)	0.083
Direct stenting after thrombus aspiration	5 (31.3%)	3 (18.8%)	0.414
Number of implanted stents in the IRA			
0	1 (6.3%)	1 (6.3%)	1.000
1	11(68.8%)	9 (56.3%)	0.716
2	4 (25.0%)	6 (37.5%)	0.704
Length of the stented segment, mm	24 [IQR 18–41]	30 [IQR 22–48]	0.202
Diameter of the stented segment, mm	3 [IQR 3–3.5]	3 [IQR 2.5–3.5]	0.651
Postdilatation	11 (68.8%)	10 (62.5%)	1.000
Intracoronary administration			
Adenosine	11 (68.8%)	12 (75.0%)	1.000
Nitrates	0	16 (100.0%)	-
Abciximab (0.25 mg/kg)	0	2 (12.5%)	0.913

Abbreviations: IRA = infarct related artery; IV = intravenous; IU = international units.

**Table 3:** Periprocedural complications.

	SALINE (n = 16)	SCT (n = 16)	*p*-Value
Transient AV-block not requiring pacing	4 (25.0%)	2 (12.5%)	0. 654
AV-block requiring pacing for drug infusion	0	0	-
Transient hypotension not requiring IABP	2	2	1.000
Hypotension requiring IABP	3 (18.8%)	2 (12.5%)	1.000
Ventricular tachycardia	0 (0%)	1 (6.3%)	1.000

Abbreviations: AV = atrio-ventricular; CV = cardiovascular; IABP: intra-aortic balloon pump; SCT = standard care of therapy.

**Table 4:** Primary and secondary endpoints and clinical outcomes.

	SALINE (n = 16)	SCT (n = 16)	*p*-Value
STR > 70%	12 (75.0%)	3 (18.8%)	0.004
Final TIMI flow 3 with MBG ˃ 1	14 (87.5%)	7 (43.8%)	0.023
Final TIMI flow 3 with MBG ≤ 1 or TIMI ˂ 3	2 (12.6%)	9 (56.3%)	-
Combined STR and angiographic MVO resolution	10 (62.5%)	1 (6.3%)	<0.001
LVEF at discharge, %	52.9 ± 8.8	55.8 ± 11.4	0.478
Hs-TnI (ng/L) peak	76.821 ± 69.402 [IQR 29544, 86250]	79.989 ± 78.261 [IQR 29932, 133549]	0.720
MACCE at 3 years	1 (6.3%)	8 (50.0%)	0.047
Cardiovascular death	0	0	
MI	0	1 (6.3%)	
TLR	0	2 (12.5%)	
HF hospitalization	1 (6.3%)	3 (18.8%)	
CVE	0	2 (12.5%)	
LVEF Δ* at 1 year	+6.8 ± 8.5	+5.6 ± 11.4	0.728
Any Bleeding	0	1 (6.3%)	-

* = considering the difference between values at baseline and 1-year follow-up. Abbreviations: CVE = cerebro-vascular events; HF = heart failure; Hs-TnI: high-sensitivity troponin I; LVEF = left ventricular ejection fraction; MACE = major adverse cardiac events; MACCE = major adverse cardiac and cerebro-vascular events; MBG = myocardial blush grade; MI = myocardial infarction; MVO = microvascular obstruction; pPCI = percutaneous primary coronary intervention; MBG = myocardial blush grade; SCT = standard care of therapy; STR: ST resolution; TIMI = thrombolysis in myocardial infarction; TLR = target lesion revascularization.

## Data Availability

The data that support the findings of this study are available from the corresponding author LG, upon reasonable request.
